# Histochemical Study of the Rat Uterine Glycoconjugate Alteration following Treatment with Exogenous Gonadotropic Hormones during the Implantation Period

**DOI:** 10.1155/2020/3967427

**Published:** 2020-12-04

**Authors:** Elham Aliabadi, Zohreh Makoolati, Tahereh Talaei-Khozani, Fakhreddin Mesbah Ardekani, Arvin Aliabadi

**Affiliations:** ^1^Department of Anatomical Sciences, Shiraz University of Medical Sciences, Shiraz, Iran; ^2^Department of Anatomical Sciences, Fasa University of Medical Sciences, Fasa, Iran; ^3^School of Veterinary Medicine, Kazerun Branch of Islamic Azad University, Kazerun, Iran

## Abstract

One of the female causes of infertility is anovulation which is treatable with gonadotropin hormones. These hormones affect the molecular organization of the uterus such as glycoconjugates that are the first site of contact between the blastocyst and the uterus. The objective of this project was to study the alteration of glycoconjugates on the uterine apical, Golgi zone, and basement membrane of epithelial cells and the uterine gland after hyperstimulation with pregnant mare serum gonadotropin (PMSG) (4, 8, 16, 24, and 40 IU), during the implantation period. Injection of PMSG (in experimental groups) and injection of distilled water (in the control group) were followed by HCG administration (10 IU), mating, isolation of positive vaginal plug rats, and killing at 5.5 days of pregnancy. Histochemistry was done on the pregnant uterine horns with the use of WGA, DBA, PNA, ConA, SBA, and UEA lectins. The intensity of the immunohistochemical staining was scored, and quantitative data were generated. 4 IU did not show any significant differences with the control, 8 IU had less effect on the alteration of the Golgi zone, and apical and basement membrane glycoconjugates and 40 IU had the least effects on the alteration of uterine gland glycoconjugates. Also, 24 IU had the most effect on the alteration of uterine glycoconjugates. Understanding of the effects of gonadotropin hormones at the uterine level in implantation time helps to optimize hormonal manipulation for improving the outcome of assisted reproductive procedures. It seems that the optimal dose for superovulation and less alteration in uterine glycoconjugates of rats at implantation time were induced by the administration of 8 IU PMSG.

## 1. Introduction

Infertility is a global health issue and affects about 10-15% of reproductive-age couples. The etiologies of infertility are male and female factors [1]. One cause of infertility is anovulation which is the most common etiology of female infertility [2, 3]. This problem is treatable with the use of gonadotropin hormones in the process of in vitro fertilization (IVF) that result in the formation of supernumerary oocytes [4]. However, in addition to superovulation, these hormones contribute to the inhibition of blastocyst implantation through its effect on the carbohydrates in the glycocalyx of endometrial cells around the time of implantation [5, 6]. The process of implantation is a highly coordinated interaction in which the embryonic trophectoderm/trophoblast establishes contact and invades the endometrial lining of the maternal uterus [7]. Before implantation can occur, the embryo must attach to the uterine tissue and the uterus must be receptive to the embryo. Since the glycocalyx of the endometrium is the first site of contact between the blastocyst and the uterus, it is possible that carbohydrates in the glycocalyx play a role in the attachment and subsequent implantation of the embryo [5]. During early pregnancy and particularly during implantation, changes in the molecular organization of the uterine epithelial cells occur [7]. It is known that cell-cell interactions are mediated by surface glycoproteins and the glycocalyx of the surface epithelium [8]. Certain carbohydrates such as galactosamine (GalNac), fucose (Fuc), glucosamine (GlcNac), galactose (Gal), and mannose (Man) in the glycocalyx of surface epithelial cells are involved in the attachment and implantation of the blastocyst [9–11]. The abnormal morphology of the surface epithelium and the disruption of the glycocalyx following factors such as stress and hyperstimulation have significant implications for the success of embryo attachment and implantation [7, 12]. These factors may warrant further investigation in attempting to explain why IVF has a low success rate [13]. The modulation of carbohydrates that result in the administration of gonadotropin hormones is evaluated with lectins that are proteins of nonimmune origin with a high degree of specificity binding ability to carbohydrates [14].

Due to the ethical restriction and experimental limitation, it is difficult to generate such information in humans [15]. Thus, the effect of pregnant mare serum gonadotropin (PMSG) on specific carbohydrates in the glycocalyx of the rat endometrium around the time of implantation was investigated.

## 2. Materials and Methods

### 2.1. Planning and Designing Animal Experiments

Five groups (experimental animals in the four groups and one control group) with 8 rats in each of them were used for this experiment. Female Sprague-Dawley rats with the average age of 60 days were obtained from the animal unit of Shiraz Medical University at a body weight of 200-250 g. They were allowed free access to food and water and were maintained on a 12 h light : 12 h dark cycle [5]. The Ethics Committee of the College of Shiraz University of Medical Sciences approved the study. All efforts were made to minimize the suffering of the animals, and the pregnant rats were euthanized at day 5.5 of pregnancy under deep anesthesia by diethyl ether (Merck, Germany). All experiments were performed in accordance with relevant guidelines and regulations.

Rats of experimental groups received an intraperitoneal (i.p.) injection of 4, 8, 16, 24, or 40 IU PMSG (purchased from NASR Pharmaceutical Co., Iran) followed by 10 IU human chorionic gonadotropin (HCG) (purchased from Darou Pakhsh, Iran) 48 h later. The female rats were then mated with proven fertile male rats. Mating was established by the presence of a vaginal plug. The day of mating was termed as day 0.5 of pregnancy. The animals were killed on day 5.5 of pregnancy (day of implantation), and both uterine horns were removed from each animal [5].

In the control group, daily vaginal smears were taken from female rats in this group. The rats were injected intraperitoneally with distilled water at diestrus or proestrus phases and then 10 IU HCG at the estrus phase. Female rats were then mated with proven fertile male rats and killed at 5.5 days of pregnancy [12]. Both uterine horns were removed.

### 2.2. Establishment of Pregnancy

**Figure 1:**
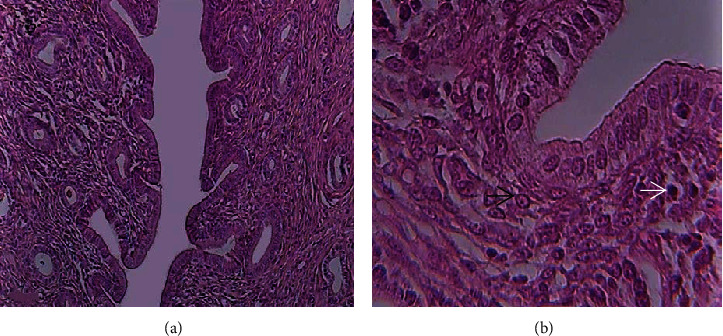
Pregnant rat uterine sections of decidual area invagination (a) and reaction (b). The *white arrow* indicates decidual reaction and the *black arrow* shows the presence of immune system cells. Magnification: (a): 20 and (b): 40.

The uterine horns were fixed in Bouin's fluid. After normal tissue preparation, uterine horns were embedded in paraffin wax. The right uterine horns were stained with hematoxylin and eosin and viewed with a light microscope. The pregnancy symptoms (i.e., invagination of the decidual area to create a pocket for the blastocyst [16]), decidual reaction [16], and presence of immune system cells [17] were investigated (Figure 1). Only animals with typical pregnancy signs were selected for lectin histochemistry on their left uterine horns.

### 2.3. Lectin Histochemistry

Lectin staining was performed as previously described [12]. Briefly, left pregnant uterine horn sections were mounted on poly-L-lysine coated slides, cleared, rehydrated through graded alcohols, and washed with 0.1 M phosphate-buffered saline (PBS). Tissue sections were treated with 1% H_2_O_2_ in methanol for 20 min to block the endogenous peroxidase and rinsed in PBS. Sections were incubated with peroxidase conjugated lectins for 2 h at room temperature. The lectins were used at 10 *μ*g/ml in PBS and included WGA (wheat germ agglutinin), PNA (peanut agglutinin), DBA (Dolichos biflorus agglutinin), ConA (concanavalin A), SBA (glycine max or soybean) and UEA (Ulex europaeus agglutinin). These lectins investigate sialic acid/N-acetylglucosamine (GlcNac), galactose/N-acetylgalactosamine (Gal/GalNac), GalNac, mannose (Man), GalNac, and fucose (Fuc), respectively [18–21]. After washing in PBS for 45 min, incubation of sections with DAB/H_2_O_2_ solution (0.03 g DAB/100 ml PBS containing 200 *μ*l of H_2_O_2_) were done for 10 min. Sections were washed with tap water for 45 min and counterstained with aniline blue for 30 sec. After washing with deionized water and dehydration through graded alcohols, sections were mounted. Control sections were incubated in PBS at room temperature.

The intensity of the positive staining in the apical membrane, Golgi zone, and basement membrane of endometrial cells and also uterine glands was determined based on an arbitrary scale by Gong et al. from 0 to 4 (no reaction to strong) [22]. This evaluation was done two times a week blindly.

### 2.4. Data Analysis

Data were analyzed statistically by Kruskal-Wallis and a nonparametric test of Mann-Whitney with the SPSS program (version 12.0) package. The significance level was *p* < 0.05 and *p* < 0.002 for the Kruskal-Wallis and Mann-Whitney tests, respectively.

## 3. Results

The intensity of the lectin reactions in the apical membrane, Golgi zone, and basement membrane of the endometrium and uterine glands was compared, and the following results were found:

### 3.1. Lectin Reactivity of Apical Membrane Glycoconjugates

The apical endometrial surface of the control group reacted with all lectins with minimum intensity for PNA and maximum for ConA.

**Figure 2:**
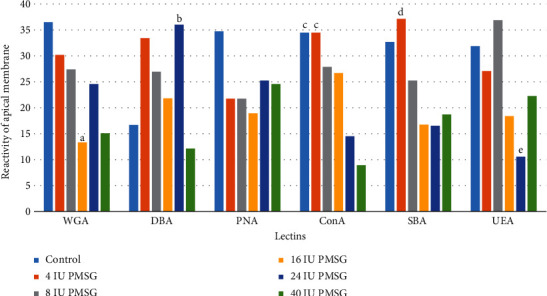
The comparison (mean rank) between the intensity of the apical membrane of endometrial cell reaction to different lectins in experimental (4, 8, 16, 24, and 40 IU PMSG) and control (distilled water+HCG) groups. ^a^Significant difference with control (*p* = 0.001). ^b^Significant difference with 40 IU and control (*p* = 0.001). ^c^Significant difference with 24 and 40 IU (*p* = 0.001 and 0.0001, respectively). ^d^Significant difference with 16 IU (*p* = 0.001). ^e^Significant difference with 8 IU and control (*p* = 0.0006 and 0.0001, respectively).

**Figure 3:**
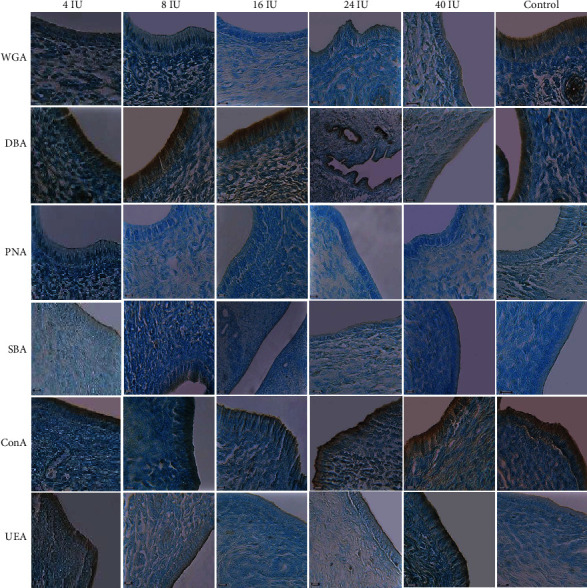
Intensity of the reaction of rat endometrium in apical membrane, Golgi zone, and basement membrane to different lectins in experimental (4, 8, 16, 24, and 40 IU PMSG) and control (distilled water+HCG) groups. Significant differences in the intensity of the reaction to different lectins were as follows: to WGA between 16 IU and control (*p* = 0.001) in the apical membrane and Golgi zone, to DBA between 24 IU with 40 IU and control (*p* = 0.001) in the apical membrane, between 24 IU with 16 IU, 40 IU and control (*p* = 0.001, 0.0001, and 0.001, respectively) in the Golgi zone, and between 4 IU with 16 and 40 IU in the basement membrane (*p* = 0.001 and 0.0001, respectively), to ConA between 4 IU and control with 24 and 40 IU (*p* = 0.001 and 0.0001, respectively) in the apical membrane and the Golgi zone, to SBA between 4 IU and 16 IU (*p* = 0.001) in the apical membrane and between control with 8, 16, and 24 IU (*p* = 0.1, 0.1, and 0.01, respectively) in the basement membrane and to UEA between 24 IU with 8 IU and control in the apical membrane (*p* = 0.0006 and 0.0001, respectively) and Golgi zone (*p* = 0.0001 and 0.0006, respectively).

The intensity of the reactions to WGA in 16 IU significantly decreased compared with that of the control group (*p* = 0.001). DBA-reacted sugars of 24 IU of the PMSG group showed a significant increase compared to the 40 IU of the PMSG and control groups (*p* = 0.001). Reactions to ConA in 24 and 40 IU of PMSG groups significantly decreased compared with that of 4 IU and control groups (*p* = 0.001 and 0.0001, respectively). The intensity of the reaction to SBA of 16 IU of PMSG significantly decreased when compared with that of 4 IU of PMSG (*p* = 0.001) and to UEA of 24 IU of PMSG significantly decreased when compared with that of 8 IU of PMSG and control groups (*p* = 0.0006 and 0.0001, respectively; Figures 2 and 3).

### 3.2. Lectin Reactivity of Golgi Zone Glycoconjugates

**Figure 4:**
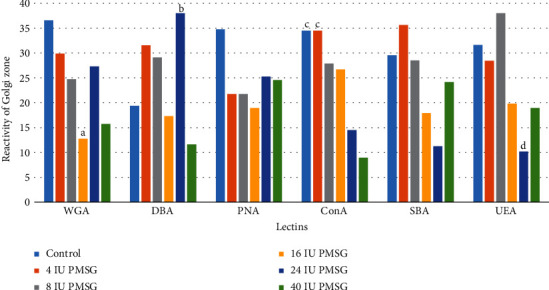
Intensity of the Golgi zone of endometrial cell reaction (mean rank) to different lectins in experimental (4, 8, 16, 24, and 40 IU PMSG) and control (distilled water+HCG) groups. ^a^Significant difference with control (*p* = 0.001). ^b^Significant difference with 16 IU, 40 IU, and control (*p* = 0.001, 0.0001, and 0.001, respectively). ^c^Significant difference with 24 and 40 IU (*p* = 0.001 and 0.0001, respectively). ^d^Significant difference with control and 8 IU (*p* = 0.0006 and 0.0001, respectively).

The Golgi zone of the control group reacted with all lectins with minimum intensity for PNA and maximum for ConA. The intensity of the reactions to WGA in 16 IU significantly decreased compared with that of control group (*p* = 0.001). Significant increase was observed in the intensity of the reaction to DBA between 24 IU with 16 IU, 40 IU, and control groups (*p* = 0.001, 0.0001, and 0.001, respectively). Reactions to ConA in 24 and 40 IU of PMSG groups significantly decreased compared with those of 4 IU and control groups (*p* = 0.001 and 0.0001, respectively). Also, significant decrease was observed in the intensity of the reaction to UEA between 24 IU with 8 IU of PMSG concentration and control groups (*p* = 0.0001 and 0.0006, respectively; Figures 4 and 3).

### 3.3. Lectin Reactivity of Basement Membrane Glycoconjugates

**Figure 5:**
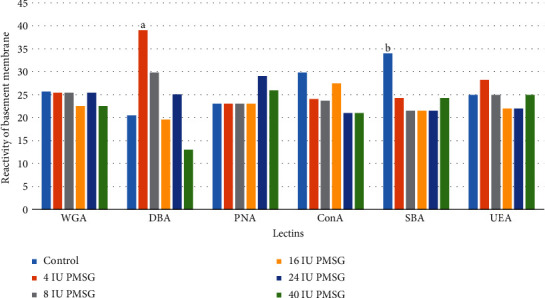
The intensity (mean rank) of the basement membrane of endometrial cell reaction to different lectins in experimental (4, 8, 16, 24, and 40 IU PMSG) and control (distilled water+HCG) groups. ^a^Significant difference with 16 and 40 IU (*p* = 0.001 and 0.0001, respectively). ^b^Significant difference with 8, 16, and 24 IU (*p* = 0.1, 0.1, and 0.01, respectively).

The basement membrane of the control group showed no reaction in the uptake of the PNA lectin. In contrast, maximum intensity was seen for SBA so that the intensity of the basement membrane reaction to SBA in 8, 16, and 24 IU of PMSG significantly decreased relative to that of the control group (*p* = 0.1, 0.1, and 0.01, respectively). Reaction to DBA in 16 and 40 IU of PMSG groups significantly decreased compared with that in the 4 IU concentration (*p* = 0.001 and 0.0001, respectively; Figures 5 and 3).

### 3.4. Lectin Reactivity of Uterine Gland Glycoconjugates

**Figure 6:**
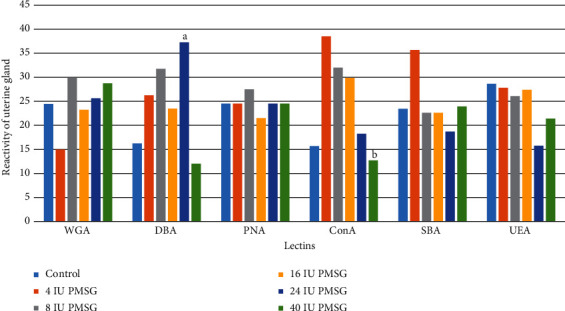
The comparison (mean rank) of the uterine gland reaction to different lectins in experimental (4, 8, 16, 24, and 40 IU PMSG) and control (distilled water+HCG) groups. ^a^Significant difference with 40 IU PMSG and control groups (*p* = 0.0006 and 0.001, respectively). ^b^Significant difference with 4 and 8 IU of PMSG (*p* = 0.0003 and 0.0001, respectively).

**Figure 7:**
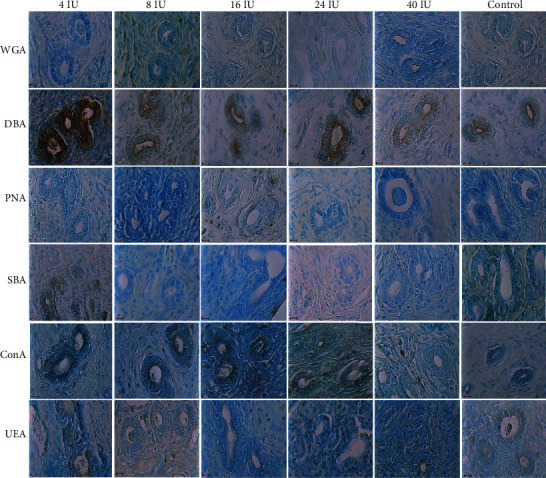
Intensity of rat uterine gland reaction to different lectins in experimental (4, 8, 16, 24, and 40 IU PMSG) and control (distilled water+HCG) groups. Significant difference in DBA-reacted sugars of 24 IU with 40 IU and control groups (*p* = 0.0006 and 0.001, respectively) and in ConA-reacted sugars of 40 with 4 and 8 IU of PMSG (*p* = 0.0003 and 0.0001, respectively) was observed.

The results obtained from the control group of the uterine gland indicated that the uterine epithelium could uptake PNA with the lowest and DBA with the highest intensities. Glycocalyx of uterine glands reacted significantly with DBA in 24 IU in comparison with 40 IU of PMSG and control groups (*p* = 0.0006 and 0.001, respectively). Also, in the uterine glands, significant decrease was observed in the intensity of the reaction to ConA of 40 IU compared to 4 and 8 IU of PMSG (*p* = 0.003 and *p* = 0.0001, respectively; Figures 6 and 7).

## 4. Discussions

This study was performed to examine the effects of hyperstimulation on the glycoconjugates of the rat endometrium. Data from the present study showed that PMSG in the 24 IU concentration had the most effects on the alteration of glycocalyx on the apical epithelial cell membrane, Golgi zone, and basement membrane of endometrial cells and uterine glands. In comparison with the control group, 4 IU did not show any significant differences, and the less effect on the alteration of glycoconjugates in the apical membrane, Golgi zone, and basement membrane of endometrial cells belongs to the 8 IU PMSG. Also, less alteration of uterine gland reactivity to WGA, DBA, and UEA was observed with 8 IU PMSG and to PNA, ConA, and SBA with the 40 IU PMSG concentration. Understanding the effects of gonadotropin hormones at the uterine level in implantation time enables manipulation of uterine receptivity to control fertility and to improve the outcome of assisted reproductive procedures [23]. Superovulatory doses of gonadotropins result in reduced fertility in the laboratory [24]. These hormones affect the glycocalyx of the endometrium. Disruption of the glycocalyx following hyperstimulation has significant implications for the success of embryo attachment and implantation [25]. Our results showed that 4 IU did not show any significant differences with the control; it can be suggested that this dose of hormone can be used as a control group in the next studies of all studied glycoconjugates.

In this study, the epithelium of the control group reacted with WGA during the implantation period, while the reactions of the apical epithelial cell membrane and the Golgi zone to WGA in treated groups decreased. This finding is in agreement with the previous studies which showed that WGA-reacted residues increase on the rat endometrial surface during implantation [9]. During embryonic development, GlcNac mediate cell-cell and cell-matrix interaction [26]. So, it may show the recognition site for embryo implantation [27]. We observed that most effects of hyperstimulation are on the glycoconjugates of the apical membrane and Golgi zone of endometrial cells. Reduction in the glycocalyx of the hyperstimulated animals including galactosamine, fucose, and glucosamine monosaccharides and trisaccharides at the time of implantation was reported in previous studies [5, 28], while in 1991, Murphy and Turner demonstrated that the same carbohydrates of the uterine epithelial cell glycocalyx increase in normal pregnant rats at implantation day [9].

These carbohydrates play a role in endometrial development that is necessary for nidation [29], initiation of blastocyst adhesion to the endometrium [30], and cell-cell or cell-matrix interaction during embryonic development [26]. Alterations in the distribution of these glycoconjugates followed by hyperstimulation cause uterine functional abnormalities. As such, no implanting embryos have been reported in animals treated with this method [28]. Therefore, we should use from those concentrations of gonadotropin hormones that have the less effects on the alteration of uterine glycoconjugates at the time of implantation to improve the success rate of IVF.

Another possible explanation for implantation failure following hyperstimulation might be due to the pathological morphologic changes in the endometrium, e.g., early increase in the epithelium height of the surface and gland, increase in the number and length of microvilli, and decrease in the mitotic activity of surface epithelium and stromal cells, which adversely affect embryo attachment and implantation [28, 31]. Adverse effects of exogenous gonadotrophins on the rat and mouse endometrium at the implantation window in a dose-dependent manner was also reported by previous studies [32–34].

The basement membrane of the control group showed maximum intensity in the uptake of the SBA lectin. Implantation consists of three stages: apposition, adhesion (attachment), and invasion [35, 36]. It is possible that carbohydrates in the glycocalyx of the basement membrane play a role in the subsequent implantation of the embryo [5]. Thus, it is rational to propose that the PMSG can reduce the receptivity of the endometrium via reduction of GalNac during the implantation period. The basement membrane of the control group also reacted with other lectins except PNA. UEA- and WGA-reacted carbohydrates were involved in cell invasion and cell-cell interaction, respectively [26, 37].

Findings of uterine glands showed positive reaction to all lectins used with the most reactivity to DBA and the least reactivity with PNA. Reaction of rat uterine glands with WGA, DBA, ConA, and UEA at days 10, 12, and 15 of pregnancy was reported previously [38]. The data showed that these glycocalyx remains exist from the implantation start time, while it seems that PNA-reacted sugars express after implantation as Lee and Damjanov showed that PNA reacted only with some human uterine glands in the proliferative endometrium while it bound intensely to all glands in the endometrium of pregnancy [39]. Thus, it seems that the PNA expression pattern in rat uterine glands is similar as that in humans. Since uterine glands through their secretions can support the growth and development of the embryo at the peri-implantation period [39], any alteration in the glycocalyx expression can affect the blastocyst implantation indirectly.

At the time of implantation, most rat uterine gland reactivity was seen with WGA, DBA, and UEA, and 8 IU PMSG has the least effects on the alteration of these lectin reactivities in the uterine gland, and all the glycoconjugates were studied in this investigation at the apical membrane, Golgi zone, and basement membrane of endometrial cells. Thus, it seems that 8 IU PMSG is the best concentration among the different doses of PMSG that were studied in this research. Based on the study by Fischer and Fisher [40], the best superovulatory doses of PMSG on the rate and time of ovulation and ovarian histology in gerbils was observed with 10 IU PMSG. Based on the above consideration, we concluded that the optimal dose for superovulation and less alteration in uterine glycoconjugates of rats at implantation time was induced by the administration of 8-10 IU PMSG.

## Data Availability

The data used to support the findings of this study are available from the corresponding author upon request.

## Abbreviations

WGA: Wheat germ agglutinin
DBA: Dolichos biflorus agglutinin
PNA: Peanut agglutinin
ConA: Concanavalin A
SBA: Soybean agglutinin
UEA: Ulex europaeus agglutinin
PMSG: Pregnant mare serum gonadotropin
HCG: Human chorionic gonadotropin
GalNac: Galactosamine
Fuc: Fucose
GlcNac: Glucosamine
Gal: Galactose
Man: Mannose
IU: International unit
IVF: In vitro fertilization
i.p.: Intraperitoneal.
